# Supplementary material for: Are carbon emissions trading and green financial instruments synergistic? -Comprehensive quantitative research based on content analysis
Source: PLoS One. 2024 Mar 7;19(3):e0298601. doi: 10.1371/journal.pone.0298601 (PMC10919601; doi:10.1371/journal.pone.0298601)
Supplement: S1 Data — (ZIP) [file pone.0298601.s001.zip › Data/地级市Z_F工作报告.zip!/┴╔─■/╜⌡╓▌╩╨/└┤╫╘╣1⁄2╓┌║┼í╛┬φ┐╦╩2╛▌═°í┐_2015.docx]

各位代表：

　　现在，我代表市政府向大会报告工作，请予审议，并请市政协各位委员提出意见。

　　一、2014年工作回顾

　　过去的一年，面对严峻复杂的经济形势和艰巨繁重的工作任务，在省委、省政府和市委的正确领导下，我们团结依靠全市人民，统筹推进稳增长、促改革、调结构、惠民生各项工作，经济社会发展取得新的成就。

　　全市实现地区生产总值1377.8亿元，增长6.2%；固定资产投资971亿元，增长2%；一般公共预算收入136.8亿元，增长0.6%；社会消费品零售总额555亿元，增长12.4%；城镇居民人均可支配收入25214元，农村居民人均可支配收入11723元，均增长10.1%。

　　(一)狠抓经济运行，稳增长扎实有效

　　工业经济稳定增长。落实市领导包扶重点企业、支持民营经济发展、举办地工产品产销衔接会等政策措施，切实帮助企业解决实际问题。锦州石化280万吨柴油加氢、天龙20万吨差别化纤维等84个重点项目竣工投产。城市分布式建筑光伏电站示范项目通过省验收。5个省重点产业集群完成销售收入1271亿元，增长6.5%。主营业务收入10亿元以上企业达到42户。实施企业并购项目4个。规模以上工业增加值增长5.4%。

　　服务业拉动作用增强。服务业发展四年行动计划全面实施。中央十里商街现代服务业集聚区建设步伐加快，北普陀山文化休闲旅游集聚区获评省级服务业集聚区。6个省级集聚区实现主营业务收入385亿元，增长20%。“夜经济”持续升温，“老锦州食坊”和“凌河美食街”建设全面启动。批发零售业销售额、住宿餐饮业营业额分别增长19.6%和15.7%。完成房地产投资201亿元，商品房销售面积385万平方米、销售额160亿元。设立首个电子商务产业基地，电子商务实现交易额22.5亿元。会展业实现交易额50亿元，增长20%。温泉、沟域、乡村旅游快速发展，北镇观音阁和青岩寺景区晋升为国家AAAA级景区。旅游业总收入实现275.8亿元，增长14.3%。成功举办辽西区域金融中心建设座谈会，盛京银行锦州分行正式营业，辽宁精细化工公司在香港上市。全年新增贷款149亿元、非信贷融资90亿元。完成个体户转企升级2828户，54户工业企业完成主辅分离。

　　重大基础设施建设稳步推进。锦州湾机场主体工程竣工，锦凌水库如期蓄水，滨海大道锦州段续建工程、机场1号路A段、中央南街立交桥、滨河路二期主体工程等建成通车，龙栖湾港、滨海体育中心体育场、锦凌水库净水厂、士英街桥洞拓宽改造等工程顺利推进。

　　对外开放势头良好。落实主体责任，县(市)区招商引资主动性明显增强。圆满承办第十三届“辽宁(锦州)台湾周”，央企、民企、沪企辽宁行和港台、北京、上海等地招商活动成效明显，上海复星生物医药、新光台湾工业园等一批项目成功签约。落实出口扶持政策，新增自营进出口权企业58家，出口基地建设进一步加强。全市实际利用外资12.5亿美元，引进省外资金342亿元，均增长10%；外贸出口24亿美元，增长13.7%。

　　锦州石化、中信锦州金属、锦州港、锦州银行等企业为全市稳增长做出重要贡献。

　　(二)创新体制机制，促改革实现突破

　　大力简政放权。完成第六次行政审批制度改革，取消、下放、调整、合并行政审批事项212项，取消行政审批前置要件80项，清理非行政许可审批11项。优化建设工程项目审批流程，变串联审批为并联审批，审批时限由275个工作日压缩到40个工作日。新公共行政服务中心主体工程完工。城市规划管理行政处罚权以外的9项城市管理权限全部下放城区。

　　机构改革卓有成效。整合经济技术开发区、龙栖湾新区、建业经济区，组建锦州滨海新区。完善食药监、工商、质监管理体制，整合卫生和计生、城乡建设和公用事业与房产管理等相关机构。成立市委市政府督查室，建立大督查体制。通过改革共精简政府工作部门7个、撤销事业局和单位5个、减少内设机构59个，137家事业单位完成隶属关系调整。制约发展的体制机制障碍进一步破除，政府职能转变步伐不断加快。

　　国有资产整合与重组工作稳步实施。15户全民所有制企业公司化改造基本完成，国有文艺院团转企改制及整合工作深入推进，新华书店转企改制、锦州广播电视报改革工作全面完成。林权制度改革、农村土地承包经营权确权登记进展顺利。工商登记制度改革加快推进，新发展各类市场主体2.2万户。

　　坚持有效防控债务风险。调整完善市规划委员会职责，整合市土地储备中心，成立市土地储备委员会、投融资管理领导小组，规划审批、土地收储、投融资工作进一步规范，财政管理工作全面加强。

　　(三)推进转型升级，调结构步伐加快

　　产业结构进一步优化，三次产业结构调整为14.6：46.7：38.7。

　　创新对产业升级的引领作用日益增强。国家硅材料及光伏高新技术产业化基地、国家火炬锦州汽车零部件特色产业基地分别实现销售收入108亿元和140亿元，规模以上工业企业完成高新技术产品增加值313亿元。新认定高新技术企业9家，引进海外研发团队项目11项，省级重点实验室、省级工程技术研究中心达到35家。成功举办“高校(科研院所)锦州行”活动，成立4个院士工作站，市政府与18家高校(科研院所)签订合作协议，开启了产学研用协同创新发展的新篇章。

　　沿海、县域、城区三大经济区域协调互动发展。锦州港货物吞吐量突破亿吨。滨海新区嘉合化工项目实现销售收入20亿元，中北石化、海森堡石化等项目即将投产，365客服基地主体工程完工，城中村改造步伐加快，大学城建设全面启动。大有格兰德新能源等项目开工建设。县域重点工业园区建设势头良好，新上固投亿元以上项目10个，北镇工业炉及配套、义县新型冶金材料两大产业集群销售收入分别突破100亿元和200亿元。“一县一业”不断壮大。黑山在国家工商总局成功注册“物产黑山”商标，“黑山褐壳鸡蛋”畅销全国。凌海成为全省唯一一家国家农业(生猪)标准化示范县。积极应对罕见旱灾，粮食产量达到38亿斤。新增设施农业10万亩、灌溉面积16.8万亩，新建国家级粮油高产示范区64个。农业产业化龙头企业达到680家，农民合作社达到3172个，农业综合机械化水平达到78%。城镇化建设步伐加快，城中村、城边村改造稳步推进。城区简政放权激发发展活力，“两项改革”成效显著，城区经济日趋活跃。古塔区石化产业园空间规划、拆动迁等前期工作全面展开。凌河区百脑汇IT总部经济大厦、茂业百货、银河广场等重大服务业项目顺利推进。太和区以粮食物流产业园为牵动的大宗商品物流业规划建设全面启动。松山高新区晋升国家级高新区工作取得实质性突破，兴隆大家庭、国际五金机电城等项目开业运营。

　　(四)注重生态建设，城乡环境不断改善

　　统筹推进碧水、青山、蓝天工程。“两河”治理工程完成总任务70%，太和区亮甲河治理工程初见成效，大凌河义县城区段二期绿化和凌海城区段河道生态工程竣工，退田还河生态封育18万亩。城市污水处理率达到85%。完成环西北、锦州城北和沿海防护林带26.4万亩，实施青山围栏封育100公里、闭坑矿山治理738亩、水土流失治理32万亩，建成千村绿化示范村35个。新热源厂开工建设，取缔燃煤小锅炉55台，购置新能源公交车125台。

　　强力推进市容环境整治半年行动。取缔客运人力三轮车4438台，拆除各类违章建筑2.5万处，重新硬化绿化63万平方米，打造示范街路20条。完成老旧小区综合改造200万平方米。儿童公园改造工程竣工。新建停车场14个，施划临时停车泊位2.7万个，城区车辆静态管理全面实施。城市网格化管理机制初步建立。网吧、小饭桌食品安全、禁限燃放烟花爆竹、学校及医院周边环境等专项整治成效明显，群众反映强烈的城市顽症得到有效治理。

　　农村环境治理“半年会战”任务圆满完成，村庄绿化美化亮化、垃圾收集和处理设施建设、道路及边沟整治等工作全面展开，宜居乡村建设迈出新的步伐。

　　(五)办好民生实事，惠民生成效明显

　　城镇实名制就业4.2万人，登记失业率控制在3.4%以内。完成城乡低保对象重新核查认定，清退不合规低保2.3万人，城乡低保、农村五保供养标准分别提高12%和10%。建成保障性安居工程5632套，回迁安置房4254套。黑山八道壕独立工矿区改造搬迁纳入国家首批东北振兴政策支持项目，沉陷区治理创造了10天搬迁2031户的新速度，总建筑面积12万平方米的32栋回迁楼全部封顶。新建锦凌水库移民安置点7个。6.4万农村低收入群众年人均收入达到3200元以上。6.65万农村居民饮水安全问题得到有效解决。市养老综合服务中心开工建设。新建民办养老机构18所，增加养老床位1800张。新建城市社区办公用房1.5万平方米。新建城乡公益性公墓16处。城区新增、延伸公交线路6条，建成农村客运站2个、农村公路候车亭20对。大中修县级以上公路293公里，新建村内道路456公里。凌海市村内道路建设排名全省第一。

　　完成100所义务教育学校标准化建设，新建改建公办幼儿园5所，市职教园建成投入使用。义务教育均衡发展凌河区通过国家验收，北镇市、古塔区通过省验收。北镇市基础教育强县(市)区通过省验收。77家基层医疗机构实施基本药物制度。新农合筹资标准和居民大病保险补助标准分别提高到年人均400元和320元。居民健康卡发放工作顺利实施，基层医疗机构管理信息系统基本建成。“单独二孩”政策有序放开。成功举办第六届全国鼓手大赛，组织大型广场演出100场，放映农村公益电影1.4万场。义县奉国寺申遗工作进展顺利，北镇庙古建筑群维修工程获评首届“全国十佳文物保护工程”。新建农民健身示范工程20个、城市社区健身路径100套。

　　(六)切实转变作风，政府自身建设不断加强

　　自觉接受市人大及其常委会的法律监督、工作监督和市政协民主监督，坚持重大事项向人大报告、向政协通报制度，认真听取各民主党派、工商联和无党派人士的意见建议。全年办理人大建议和政协提案388件，办复率达100%。

　　深入学习贯彻习近平总书记系列重要讲话精神，扎实开展党的群众路线教育实践活动。严格执行中央八项规定，坚决纠正“四风”问题，清理超标办公用房1.55万平方米，清退违规用车170台。强力推进机关作风、政法机关执法执纪等百日专项整治行动，全市868个单位查摆整改问题6081个，清退在编不在岗人员167名，公开处理违纪人员153人。作风建设长效机制逐步建立，执法执纪行为不断规范。开展法治锦州建设年活动，深入推进全民普法宣传教育，广大干部群众法律意识不断增强。加强党风廉政建设，严格落实“两个责任”，严肃查处了一批违法违纪案件。“五大系统”建设成效明显，行政执法监督、政务公开、廉政监察、审计监督等工作进一步加强。

　　按照市委统一部署，积极开展锦州发展大讨论，锦州建设辽西区域性中心城市的定位更加明确，新一轮振兴发展蓝图已经绘就，全市上下干事创业的正能量快速集聚。

　　严格落实安全生产主体责任，强化政府应急管理，完善社会治安防控体系，依法依规开展信访工作，狠抓食品药品安全，平安和谐锦州建设迈出新的步伐。

　　国防动员、边海防建设、人防和民兵预备役、双拥共建工作取得新进步。民族宗教、国家安全、妇女儿童、慈善救助、防震减灾、气象服务、地方志、住房公积金等工作不断加强。

　　各位代表！过去一年的成绩来之不易。这是省委、省政府和市委坚强领导的结果，是市人大、市政协和社会各界监督支持的结果，是全市上下奋力拼搏的结果。在此，我代表市政府，向全市人民，向人大代表和政协委员，向参与锦州发展建设的中省直单位，向驻锦部队和武警官兵，向社会各界和离退休老同志，向所有关心支持锦州的海内外朋友，表示衷心感谢和崇高敬意！

　　在总结工作的时候，我们也清醒地看到存在的差距和问题：一是经济下行压力加大，投资动力不足，部分主要经济指标没能完成预期目标，一些既定的城建项目没有开工建设。二是区域发展不平衡，结构调整步伐不快，县域经济增长乏力，服务业特别是大宗商品物流业和现代服务业发展相对滞后。三是经济总量偏小、质量不高，缺少具有牵动作用的重大项目，一般公共预算收入增长放缓，政府债务进入偿还高峰期，收支矛盾突出。四是滨海新区和松山新区先导示范作用不强，开放度不高，创新创造活力不足。五是教育、医疗、文化等社会事业欠账较多，城市基础设施建设投入不足，民生保障压力较大。六是政府个别部门还存在不作为、慢作为的现象，行政效能需要进一步提升；腐败问题在一些行业和领域仍处于易发多发状态，党风廉政建设“一岗双责”还需进一步加强。对于这些问题，我们一定要高度重视，切实加以解决。

　　二、2015年工作安排

　　2015年是全面深化改革、全面推进依法治市的关键之年，是完成“十二五”规划的收官之年，也是深入实施新一轮东北振兴发展的开局之年。面对新常态带来的经济运行新特征、新规律和新要求，我们只有主动适应、科学把握、积极作为，才能抢占先机、赢得发展的主动。全市上下必须紧紧围绕辽西区域性中心城市建设这一宏伟目标，抢抓新一轮东北振兴机遇，开拓创新，奋发有为，继续保持经济稳中有进的良好态势，完成“十二五”规划目标任务，不断开创改革发展的新局面。

　　2015年政府工作总体要求是：全面贯彻党的十八大和十八届三中、四中全会精神，按照中央和省经济工作会议、市委十一届八次全会要求，坚持稳中求进工作总基调，准确把握并主动适应经济发展新常态，坚持以提高经济发展质量和效益为中心，以转方式调结构为主线，以改革和创新为动力，以保障和改善民生为根本，以法治建设和作风建设为保障，深入实施对外开放、创新驱动、以港兴市、工业强市、生态立市战略，努力打造实力、法治、文明、生态、幸福锦州，全面加快辽西区域性中心城市建设进程。

　　2015年全市经济社会发展主要预期目标是：地区生产总值增长6.5%，一般公共预算收入增长2.3%，固定资产投资增长6%，社会消费品零售总额增长11%，城镇居民人均可支配收入和农村居民人均可支配收入分别增长8%和10%。

　　围绕全年目标，要重点做好以下八个方面工作：

　　(一)加快滨海新区和松山新区先导区建设，打造振兴发展新引擎

　　依托国家级开发区和高新区，构建沿海开放新高地和创新发展新平台，引领提升锦州城市综合竞争力。

　　深入实施“以港兴市”战略。加大海港、空港建设力度，推动临港临空经济和港铁联运产业发展，加快形成以港兴产、以产促城、港城联动新格局。主动抢抓国家“一带一路”战略机遇，积极争取国家和省支持，全力推进锦州港作为中蒙铁路新通道出海口建设。加快推进通关便利化改革。锦州港货物吞吐量稳定在亿吨以上。加快龙栖湾港疏港铁路等集疏运体系建设，确保龙栖湾港区及临港产业建设取得新进展。锦州湾机场建成通航，机场主干路全部贯通，启动空港经济区通用航空产业园建设。推进邈矣芳烃化工等项目前期工作，加快天龙差别化纤维二期等项目建设，确保锦州滨海电子商务产业基地等项目投入运营。完成汉拿电机整体迁入滨海新区，做强做大国家火炬锦州汽车零部件特色产业基地。统筹大有经济区生态旅游、健康食品、新能源等主导产业发展，开工建设大凌河滨河路、大有经济区污水处理站，加快天沐温泉、龙海馨港等重点旅游开发项目建设。

　　进一步优化滨海新区发展空间，编制完成总体规划、城市详规和产业发展规划。加快城中村搬迁改造，推进重点园区道路、管网等基础设施和街路绿化亮化工程建设，不断完善商业、教育、医疗、文化、体育等城市服务功能。全力打造大学城，确保渤海大学滨海校区和辽宁农业经济学校建成投入使用，推进辽宁医学院、锦州师专整体搬迁，加快辽宁理工职业学院本科校区建设，谋划新的职业学校入驻职教园区，积极吸引国内知名高校来锦设立分校。

　　深入实施“创新驱动”战略。召开全市科技创新大会，研究出台支持创新创造相关政策。设立科技创新基金，引进创新型科技人才、重点领域紧缺专门人才和海外研发团队，培育高技能人才队伍。进一步加强与高校、科研院所合作，与中科院沈阳分院合作共建技术转移分中心，与驻锦高校合作共建产业协同创新中心，支持有条件企业组建国家级工程技术研究中心，加快产学研用协同创新步伐。强力推进松山新区大学科技园建设。发挥院士专家工作站作用，加快高新技术创业服务中心建设，不断提升国家级科技企业孵化器辐射带动作用。进一步强化国家硅材料及光伏高新技术产业化基地建设。新认定高新技术企业10家。开工建设光电产业园、五金机电城三期、道光廿五迁建等项目，加快推进上海复星生物制药基地等项目建设。促进工业化与信息化深度融合，积极发展云计算、物联网、卫星导航服务等产业。加大组织领导力度，建设“智慧城市”。

　　(二)深化改革扩大开放，增强经济社会发展活力

　　认真贯彻落实国务院《关于近期支持东北振兴若干重大政策举措的意见》，全面深化各项改革，加快构筑高水平开放型经济体系。

　　用足用好新一轮东北振兴政策。落实市领导包扶重大项目责任制，抓好已洽谈项目对接，加快推进北镇石墨产业园、中节能城市环保合作、上海联影医学影像中心等重大产业项目落地建设。主动对接各项支持政策，深入挖掘包装一批重大项目，争取古塔城区老工业区搬迁改造等项目纳入国家老工业基地产业布局和国家、省“十三五”规划。加强与中信、华润等央企的深度合作，力争在共建产业园、培育新兴产业、工业废弃地和老矿区环境整治、城市基础配套设施建设等方面取得明显突破。科学编制好锦州“十三五”及未来十年经济社会发展规划。

　　继续推进简政放权和行政审批制度改革。巩固行政审批制度改革成果，确保已取消、下放的行政审批事项落实到位。今年再取消和下放一批行政审批事项，坚决清理并取缔非行政许可审批事项。新公共行政服务中心建成投入使用，加快建设网上审批平台，落实“两集中、两到位”，确保部门和审批事项应进必进。实行审批事项责任清单管理，实现清单外无审批。筹备承办好全省转变职能简政放权工作现场会。

　　有序推进各相关领域改革。深化国资国企改革，加快华信集团等企业资产重组，完善现代企业制度，强化国有资产监管。推进企业股权多元化改革，大力发展混合所有制经济。进一步理顺市本级与主城区、滨海新区和松山新区财政管理体制。建立健全政府债务管理制度和风险预警处置机制。深化工商登记制度改革，加快推进“三证合一”。协调推进事业单位分类改革，强化机构编制动态管理。稳步实施公用事业改革，不断深化文化体制改革，继续推进农村土地承包经营权确权登记颁证工作和集体林权制度改革。

　　深入实施“对外开放”战略。强化招商队伍建设，创新招商引资体制机制。加大面向日韩、欧美、港澳台、北京、长三角、珠三角等国家和地区的专题招商、定向招商力度，充分利用资源优势，组织好钛及特种金属等产业链招商。加快完善基础条件，狠抓项目落地，推进央企、民企、沪企辽宁行签约项目及早开工建设。研究出台扶持政策，鼓励企业走出去收购海外研发团队和企业。充分利用国家加工贸易梯度转移重点承接地政策优势，深度参与环渤海协同发展，主动接受京津冀产业转移。承办好“第十四届辽宁台湾周”。积极推进在锦设立海关特殊监管区工作。强化重点出口企业的跟踪服务，重点扶持一批出口基地和龙头企业，力争汽车零部件、铁合金两大基地升格为省级出口基地。支持企业发展跨境电子商务。确保外贸出口增长5%。

　　(三)推动工业转型升级，提高工业经济发展质量

　　深入实施“工业强市”战略，调整存量，做优增量，稳步提升工业经济运行质量，积极培育战略性新兴产业和特色产业，加快推进工业转型升级。

　　抓好工业经济运行调度。组织企业参加地工产品产销衔接会、产品订货会和展销会，帮助企业开拓国际国内市场。用好工业经济发展专项资金。清理规范涉企行政事业收费，切实减轻企业负担。加强对100户重点企业实时监测分析，千方百计确保工业企业稳定运行。

　　继续实施工业“6921”工程。加快精细化工、钛及特种金属等产业集群发展，延长产业链条，提高产业丰厚度。大力推动新能源、新材料、生物医药、节能环保等战略性新兴产业发展。出台“实施品牌战略促进产业转型升级”政策，鼓励引导20户龙头企业和100户成长型企业争创名牌产品、中国驰名商标和省著名商标。全年新增规模以上工业企业30户，规模以上工业增加值增长7%。5个省重点产业集群销售收入增长7%以上。

　　全面加快民营经济发展。落实促进民营经济发展政策措施，创新扶持模式，着力解决企业在创办、成长、创新、税费、融资等方面存在的实际困难，壮大一批民营企业集团。鼓励全民创业，让中小微企业、“专精特新”企业茁壮成长，形成大众创业、草根创业的发展浪潮，激发市场主体活力。

　　(四)坚持“三化”同步推进，提升县域经济整体实力

　　立足比较优势，坚持差异化、特色化发展，加快推进县域新型工业化、农业现代化和城镇化进程。

　　夯实县域工业基础。鼓励和引导社会资本参与工业园区基础设施、检验检测平台和研发中心建设，不断提高项目承载能力。做强做大农机装备制造、农产品精深加工、新兴冶金材料等主导产业，加快培育壮大工业产业集群。今年每个县(市)新开工投资亿元以上项目10个，其中10亿元以上2个。

　　加速农业现代化进程。规范引导农村土地经营权有序流转，促进农业现代化生产、适度规模化经营，加快培育新型农业经营主体。新增农民合作社200家、家庭农场100家、设施农业10万亩、产业化龙头企业50家。加强农业科技创新和推广，新建粮油高产示范片区58个，完成16.8万亩节水滴灌工程。粮食产量稳定在50亿斤以上。加快发展现代渔业，推进海洋牧场建设。畜牧业规模化养殖比重达到65%以上。农业综合机械化水平达到80%。继续抓好12个10亿元以上农产品加工项目的续建投产工作。加快黑山蛋鸡、北镇葡萄、义县奶牛养殖、凌海生猪养殖等“一县一业”发展。强化耕地和基本农田保护，提高土地利用集约化程度。

　　积极稳妥推进以人为核心的新型城镇化建设。科学制定城镇化发展规划，研究推进城镇化发展政策措施，适时启动城镇化建设试点工作。着力培育和发展支撑性产业，以产业带动就业，以就业聚集人气。稳步推进教育、卫生、医疗、文化等基本公共服务常驻人口全覆盖，有序推进农业转移人口市民化，加速推动城乡一体化发展。

　　(五)提升服务业发展水平，加快城区经济发展

　　大力发展现代服务业，优化提升传统服务业，拓展壮大新兴服务业，服务业增加值占地区生产总值比重比上年提高1个百分点。

　　稳步实施服务业发展四年行动计划。以中央十里商街现代服务业集聚区建设为重点，加快推进百脑汇IT总部大厦、太阳广场等在建项目建设，确保古塔区香港乐天城市综合体、凌河区迪卡侬卖场、太和区红星美凯龙综合体、松山新区万达广场等项目开工建设。按照政府引导、市场运作、科学规划、合理布局的原则，加快大宗商品交易中心和区域性农产品批发市场建设，积极打造辽西物流中心。推进辽西区域金融中心建设，重点发展产业金融、科技金融，力争浦发、华夏等域外银行在锦设立分支机构，锦州银行等企业上市。加强担保体系建设，规范小额贷款公司发展。全年新增贷款140亿元，非信贷融资70亿元。出台促进旅游业发展政策，整合旅游资源，培育特色品牌，强化宣传促销，推动服务升级，打造具有较大影响力的旅游目的地，加快推进旅游强市建设。培育品牌展会，力争展会交易额增长20%。进一步繁荣城乡商品市场。大力发展健康养老、中介服务、文化创意、研发设计等新兴服务业。加快城中村、城边村改造，实施工业、旅游、商业、健康养老、体育等地产综合开发，保持房地产业健康发展。

　　各城区要充分利用区位、基础和比较优势，促进特色发展，逐步实施“退二进三”。古塔区要以石化、电子工业园建设为重点，同步推进休闲旅游和总部经济发展。凌河区要依托辽西小商品·古玩商贸流通集聚区和中部服务业集聚区，重点发展总部经济、金融、电商等现代服务业。太和区要重点发展大宗商品物流业和大型专业市场。

　　(六)加大建设管理力度，提升城市功能和城乡环境建设水平

　　进一步加快重大基础设施和生态环境建设，深入实施城乡环境整治，保护建设我们赖以生存的美好家园。

　　着力推进重大基础设施建设。充分利用政府和社会资本合作(PPP)等多种融资模式，加大基础设施建设投入力度。开工建设广州街小凌河大桥、中环西路、滨河路三期、科技路西段、垃圾处理场等工程。完成锦凌水库25万吨净水厂主体、士英街桥洞拓宽改造、义县外环路续建等工程。启动林西街和徐州街等断头路贯通、云飞街和人民街公铁立交桥改造、城北外环路等工程前期工作。着手城市地下管线普查和综合规划，有计划改造地下老旧管网。完成中央南街立交桥绿化工程，支持京沈客运专线建设，完成征地拆迁收尾工作。

　　深入实施“生态立市”战略。制定生态建设规划，继续推进碧水、青山、蓝天工程。高质量完成“两河”治理工程，实施锦凌水库水土保持和环境保护工程，推进凌河生态文明示范区建设。新建10万吨污水处理厂。全部完成城区截污干管建设工程，彻底解决城市污水直排问题。强化海洋生态保护，集约节约利用海域资源。突出抓好三北、沿海、沿河防护林和中央财政试点造林工程建设，完成人工造林17万亩、封山育林17万亩、闭坑矿山治理647亩、水土流失治理32万亩，建成绿化示范村50个。重点抓好清洁生产、扬尘污染控制、机动车污染防治等工作，开工建设华润锦州电厂2台66万千瓦机组项目，新建热源厂2座，取缔燃煤小锅炉28台，完成大气污染防治考核任务。

　　巩固市容环境综合整治成果。确保城市网格化管理落到实处，不断提高城市科学化、精细化、智能化管理水平。推进环境综合整治向城乡结合部和县域延伸。完成中央南街立交桥等7座桥梁亮化和5条街路线杆撤线入地工程，全面提高城区美化亮化水平。

　　扎实开展农村环境建设年活动。加快推进宜居乡村建设，大力实施“十百千宜居乡村创建工程”。突出抓好消灭“四堆”、道路畅通、排水畅通、绿化、美化、文化广场建设等工作，支持有条件的村实施生态景观改造。健全农村环境治理和管护体系，打造美丽乡村35个、宜居示范村50个、宜居达标村300个。

　　(七)切实保障和改善民生，提高人民群众生活水平

　　面对全市人民过上美好幸福生活的新期待，我们将加大投入力度，加快实施进度，带着感情和责任重点办好10个方面的民生实事。

　　——开展基础教育建设年活动。出台促进基础教育发展实施意见，深化教育改革，整合教育资源，提高教育信息化水平，大力推进义务教育均衡发展和基础教育强县(市)区建设。实施农村薄弱学校改造，大力改善办学条件。加强教师队伍建设，实施中小学班主任建设工程。大力发展公办幼儿园。促进产教融合，做强做大职业教育，探索成立职业教育集团。

　　——不断提高医疗保障水平。进一步深化医疗卫生体制改革，完善基层医疗卫生体系，完成县级公立医院综合改革。启动市中心医院综合楼和公共卫生应急中心建设。普及应用居民健康卡，推广智慧医疗，实现新农合、重大疾病保障、城乡医疗救助和大病商业保险制度“一站式”服务。新农合人均筹资标准提高到450元。继续推进省内医保异地结算平台建设。落实计划生育政策，稳定低生育水平。

　　——实施文化惠民工程。举办首届锦州群众文化节，组织惠民演出200场，实施农村公益性电影放映工程，建成社区书屋96个。开工建设文化艺术中心，市京剧院维修改造工程、万人文化广场建成投入使用。做好奉国寺申报世界文化遗产基础工作。加强文化市场规范化管理，推进文化产业园区建设。

　　——大力发展体育事业。新建农民健身路径600套，实现全市农民健身工程和社区健身路径全覆盖。加强滨海体育中心运营管理，完成体育场建设。

　　——推动创业带动就业。城镇实名制就业4.1万人，登记失业率控制在4.3%以内。大力开展就业援助，登记困难家庭高校毕业生就业率达100%，保持零就业家庭动态为零。整合人才市场和劳动力市场，建设人力资源市场。

　　——完善社会保障体系。进一步扩大社会保障覆盖面，确保各项社会保险待遇按时足额支付。提高城乡最低生活保障和农村五保对象供养标准。加快市养老综合服务中心建设，筹建第三军休服务管理中心，启动市殡仪馆新建工程。加快社会养老服务事业建设步伐，支持和鼓励社会力量参与养老事业。

　　——改善群众生活条件。实施棚户区改造3910户，推进暖房子工程。开工建设配水池等4处绿地公园。整治老旧小区237万平方米，对老旧小区连接主干路破损路面及小街小巷实施维修改造。解决6.8万农村人口饮水安全问题。

　　——做好扶贫帮困工作。确保7.76万农村低收入群体稳定脱贫。实施水库移民后期扶持项目35个。对923户贫困残疾人家庭实施无障碍改造，为贫困残疾人配发辅助器具1000件。推进社会救助体系建设试点和慈善事业发展。

　　——营造便捷出行环境。新建、大修城区街路17条，彻底消除摸黑路。新增更新公交车100辆，市区至滨海新区开通公交车。规范出租车管理。推进智能交通系统建设。实施县以上公路大中修269公里，新建村内道路400公里。建成农村公路候车亭25对。

　　——推进平安锦州建设。全面完成“六五”普法任务。扎实做好民族宗教工作。积极争创全国双拥模范城“四连冠”。深入开展社会治安综合治理，深化法律援助，坚持依法处理信访，维护社会公平正义。完善突发事件应急处置机制，严格落实安全生产监管和责任追究制度，加强食品药品安全管理，严防重特大事故发生，确保人民生命财产安全。

　　(八)全面推进依法治市，强化政府自身建设

　　打铁还需自身硬。市政府领导班子要从自身做起，真抓真管、敢抓敢管、严抓严管，努力建设职能科学、权责法定、执法严明、公开公正、廉洁高效、守法诚信的人民满意政府。

　　建设法治政府。深入开展法治锦州建设年活动，依法全面履行政府职能，坚持法无授权不可为，法定职责必须为。依法规范行政权力运行，健全重大行政决策程序，完善决策机制，推进决策科学化、民主化、法治化。自觉接受人大法律监督、工作监督，坚决执行人大及其常委会的各项决议、决定，认真办理人大议案、代表建议。积极接受政协民主监督，全力支持人民政协履行职能，认真办理政协建议案、委员提案。坚持政府联系服务代表、委员制度，拓宽沟通渠道，广泛听取社会各界意见。

　　建设高效政府。巩固拓展教育实践活动成果，深入落实“四风”整治措施，坚决纠正不作为、乱作为，坚决克服懒政、怠政，坚决惩处失职、渎职。整合优化投诉举报平台，强化重大决策政务督查，严格政府绩效考核。大力倡导“钉钉子”和踏石留印、抓铁有痕的精神，满怀发展激情，勇于担当，敢于负责，环环相扣抓落实，定一件干一件，干一件成一件，全面提高政府行政效能。

　　建设廉洁政府。严格落实党风廉政建设责任制，大力推进政府系统惩治和预防腐败体系建设。深入实施“五大系统”建设，推进公共资源交易中心管办分离，深化重点领域政务公开。强化政府重大投资跟踪审计和经济责任审计，坚决落实审计整改要求，努力推进审计监督全覆盖。完善“三公”经费预算管理和公开制度，推进公务用车制度改革。坚决做到“讲诚信、懂规矩、守纪律”，积极营造良好的政治生态和从政环境。

　　各位代表，新一轮东北振兴大幕已经拉开，机遇千载难逢，奋斗创造未来。让我们在市委的坚强领导下，齐心协力，埋头苦干，为加快辽西区域性中心城市建设、创造锦绣之州更加美好的明天而努力奋斗！
